# *Leishmania*-HIV Co-infection: Clinical Presentation and Outcomes in an Urban Area in Brazil

**DOI:** 10.1371/journal.pntd.0002816

**Published:** 2014-04-17

**Authors:** Gláucia F. Cota, Marcos R. de Sousa, Andrea Laender Pessoa de Mendonça, Allan Patrocinio, Luiza Siqueira Assunção, Sidnei Rodrigues de Faria, Ana Rabello

**Affiliations:** 1 Laboratory of Clinical Research – Centro de Pesquisas René Rachou - Fundação Oswaldo Cruz, Fiocruz, Belo Horizonte, Minas Gerais, Brazil; 2 Eduardo de Menezes Hospital – Fundação Hospitalar do Estado de Minas Gerais-FHEMIG, Belo Horizonte, Minas Gerais, Brazil; 3 Post-Graduate Program in Adult Health Sciences - Universidade Federal de Minas Gerais, Belo Horizonte, Minas Gerais, Brazil; US Food and Drug Administration, United States of America

## Abstract

**Background:**

Visceral leishmaniasis (VL) is an emerging condition affecting HIV-infected patients living in Latin America, particularly in Brazil. *Leishmania*-HIV coinfection represents a challenging diagnosis because the clinical picture of VL is similar to that of other disseminated opportunistic diseases. Additionally, coinfection is related to treatment failure, relapse and high mortality.

**Objective:**

To assess the clinical-laboratory profile and outcomes of VL-HIV-coinfected patients using a group of non HIV-infected patients diagnosed with VL during the same period as a comparator.

**Methods:**

The study was conducted at a reference center for infectious diseases in Brazil. All patients with suspected VL were evaluated in an ongoing cohort study. Confirmed cases were divided into two groups: with and without HIV coinfection. Patients were treated according to the current guidelines of the Ministry of Health of Brazil, which considers antimony as the first-choice therapy for non HIV-infected patients and recommends amphotericin B for HIV-infected patients. After treatment, all patients with CD4 counts below 350 cells/mm^3^ received secondary prophylaxis with amphotericin B.

**Results:**

Between 2011 and 2013, 168 patients with suspected VL were evaluated, of whom 90 were confirmed to have VL. In total, 51% were HIV coinfected patients (46 patients). HIV-infected patients had a lower rate of fever and splenomegaly compared with immunocompetent patients. The VL relapse rate in 6 months was 37% among HIV-infected patients, despite receiving secondary prophylaxis. The overall case-fatality rate was 6.6% (4 deaths in the HIV-infected group versus 2 deaths in the non HIV-infected group). The main risk factors for a poor outcome at 6 months after the end of treatment were HIV infection, bleeding and a previous VL episode.

**Conclusion:**

Although VL mortality rates among HIV-infected individuals are close to those observed among immunocompetent patients treated with amphotericin B, HIV coinfection is related to a low clinical response and high relapse rates within 6 months.

## Author Summary

Visceral leishmaniasis (VL) is of a higher clinical importance as an opportunistic infection in individuals infected with HIV (human immunodeficiency virus type-1) in areas where both infections are endemic. Co-infected patients classically present a chronic clinical course, with high rates of treatment failure and relapse. Differences in the clinical presentation of VL between HIV-infected and uninfected patients and the factors related to an unfavorable outcome remain rarely studied. In this work, the clinical and laboratory characteristics of patients with VL were compared according to HIV infection status, and the main determinants of a poor outcome at 6 months were identified.

## Introduction

In Latin America, the causative agent of visceral leishmaniasis (VL) is the intracellular protozoan *Leishmania infantum* (syn. *L. chagasi*). VL occurs from Mexico to Argentina but the majority of the cases in South America are reported in Brazil [1], where there has been a trend toward VL urbanization and an increased rate of co-infection with HIV in recent years [2]. Differences in the clinical presentation of VL between HIV-infected and uninfected patients and the factors related to an unfavorable outcome are scarcely studied. VL mortality is particularly high among immunosuppressed patients [3]. However, most studies of *Leishmania*-HIV coinfection have been conducted in Europe and Africa, which likely present a different scenario than in Latin America. HIV infection and several other risk factors have been related to death, such as the presence of very low counts of neutrophils and platelets, dyspnea, jaundice, mucosal bleeding and bacterial infections [4]–[7]. Death can be due to VL itself or direct drug toxicity. Moreover, both parasite and host determinants can influence the treatment failure rate [8]. This study aimed to describe the clinical picture and factors related to clinical outcomes in *Leishmania*-HIV coinfected patients at an urban referral center in Brazil.

## Methods

### Study design and participants

From February 2011 to March 2013, the patient inclusion phase of a prospective cohort study was performed at Eduardo de Menezes Hospital, Fundação Hospitalar do Estado de Minas Gerais (HEM-FHEMIG). The 100-bed hospital is a state reference center for infectious diseases in adults in Belo Horizonte, the capital of Minas Gerais state, Brazil, which contains 20 million inhabitants. The main objective of this cohort study was to evaluate the diagnostic accuracy of several tests [9] and to study the VL characteristics and prognosis of VL by comparing HIV-infected and non-infected patients through a clinical and laboratory follow-up for one year. Approval for this study was obtained from the Ethical Review Board of HEM-FHEMIG and from Centro de Pesquisas René Rachou, Fundação Oswaldo Cruz (CPqRR-FIOCRUZ). Patients were included in the study only after informed consent was obtained.

Clinical suspicion of VL was defined as fever for >14 days or splenomegaly or cytopenia. According to the study protocol, a parasitological test (bone marrow aspirate) was performed for all patients with suspected VL. Diagnostic confirmation was obtained based on parasitology (bone marrow aspiration) or serology plus observation of the clinical response following treatment. The Serological methods were the direct agglutination test (DAT), rk39 antigen based test (InBios International, Seattle, WA, USA) and the indirect fluorescent antibody test (Bio-Manguinhos, Rio de Janeiro, RJ, Brazil). Testing for HIV was performed for all patients with suspected VL, according to Brazilian guidelines [10].

The first-line drugs used to treat VL were pentavalent antimony or intravenous amphotericin B deoxycholate for four weeks. For HIV-infected patients, therapy with pentavalent antimony was avoided. The antimony used was antimoniate of N-methylglucamine (Sanofi Aventis, Rio de Janeiro - RJ, Brazil), which contains 81 mg of pentavalent antimony (Sb^V^) per mL (5 mL per ampoule) and was provided by the Brazilian Ministry of Health. The dose employed was 20 mg/kg/day of Sb^V^ (a maximum of three ampoules, on the recommendation of the Ministry of Health in Brazil) [10]. Amphotericin B is recommended as the first choice in patients under six months old or over 65 years old, in those with severe clinical manifestation and in HIV-coinfected patients [11]. Liposomal amphotericin B is restricted for patients older than 50 years, organ transplant receipts and those presenting renal dysfunction. In September 2013, the Brazilian Ministry of Health's policy was modified [12], including the use of liposomal amphotericin B for the treatment and prophylaxis of VL in HIV-co-infected patients. For amphotericin B, whether deoxycholate based (generic formulation) or liposomal (Gilead Sciences, San Dimas, CA, USA), a total dose of 20 mg/kg was used. After treatment, all patients with CD4 counts below 350 cells/mm^3^ received secondary prophylaxis every two weeks (twice monthly) with amphotericin B desoxicolato or liposomal amphotericin B if creatinine clearance was less than 50 mL/min. After September 2013, liposomal amphotericin B was extended to all coinfected patients. Prophylaxis was maintained for at least 6 months and it was discontinued when two consecutive CD4 cells counts were more than 350cells/mm^3^.

Clinical, sociodemographic and laboratory variables were recorded on a standardized form. The records were assessed by one of three medically-trained investigators and the forms were reviewed for missing data and consistency.

The disease's length was defined as the time interval between the onset of symptoms and diagnosis. Bleeding was assessed by history collection and physical examination. Information from patients about gastrointestinal bleeding, epistaxis or hemoptysis during the course of symptoms was considered as evidence of bleeding. Petechiae, ecchymosis and bleeding at sites of venipuncture were determined from medical and nursing records. Urinary bleeding was considered when more than 15 red blood cells per field were noted in a urinary sediment analysis. The presence of bacterial infection was defined as empirical antimicrobial therapy use and/or a bacteriologically confirmed diagnosis recorded in the chart. The spleen was measured at its greatest extent from the left costal margin, at the left midclavicular line, to the splenic tip. The liver was measured from the right costal margin, at the midclavicular line, in the craniocaudal direction. Splenomegaly was defined by the presence of a palpable spleen, and hepatomegaly was defined by a liver that was palpable more than 2 cm from the right costal margin.

The following aspects were considered to evaluated the clinical response on the last day of treatment: (a) fever response: the clearance of fever, (b) spleen response: a reduction of 2 cm or more in spleen palpation, (c) hemoglobin response: an increase of 2 g% or more in hemoglobin tax (d) leukocyte response: an increase of 50% or more in the leukocyte count and (e) platelet response: an increase of 50% or more in the platelet count. These criteria were chosen by consensus among researchers because they were considered unambiguously identifiable differences, taking into account the local routine. Clinical cure, assessed at 2 and 6 months after the end of treatment, was defined as meeting all the following criteria: (a) an absence of fever, (b) no hepatosplenomegaly and, (c) no hematological abnormalities. Patients who did not meet all of the above criteria were classified as cases with poor outcome. Death and VL relapse were also considered as poor outcomes. According to our local routine, a parasitological test of cure was not performed. Nonetheless, the collection of a new bone marrow aspirate was indicated to confirm VL relapse when the emergence or worsening of any VL signs or symptoms (fever, hepatosplenomegaly and cytopenia) was observed.

### Statistical analyses

Continuous variables were analyzed using unpaired Student's *t tests* for normally distributed variables and Wilcoxon tests for variables with skewed distributions. Alternatively, the variables were dichotomized into predefined categories to allow for comparison with previous studies (e.g., age, anemia and thrombocytopenia). Chi-square tests were used to compare categorical variables. All clinical, demographic and laboratory variables collected were explored in univariate analysis in relation to risk of early death. Similarly, univariate analyses of the factors associated with a poor outcome at 6 months were performed. All variables with a p value<0.20 were included in a multivariate logistic regression model, using the step-by-step backward likelihood ratio method. The Hosmer-Lemeshow [13] goodness-of-fit test was used to evaluate model fitness. Only variables showing a significant association (p<0.05) with the occurrence of a poor outcome in VL remained in the final model. The statistical analyses were performed by using SPSS version 16 and MedCalc.

## Results

Between 2011 and 2013, 168 patients with suspected VL were evaluated. In total, 53% (90 patients) were then confirmed to be VL patients, of whom 46 were HIV coinfected (51%). VL diagnosis was confirmed by a parasitological exam of bone marrow aspirate in 65 patients (65/79, 82.3%) and by serological tests plus observation of the response to therapy in 25 patients. The patients' ages ranged from 14–68 years, with a mean of 39.1±12.6 years. Overall, 68 (76%) were females. Fifteen of 46 HIV-infected patients had a diagnosis of simultaneous HIV and VL infection (33%), and 15 (33%) had presented an opportunistic infection in the past. Additionally, 30 patients (65%) were on highly active antiviral therapy (HAART), although only 35% of the total (16 patients) were taking medication regularly. The median CD4 lymphocyte count of the 42 HIV-infected patients with available information collected within 3 months before admission was 91 cell/mm^3^ (25–75%IR 39–194) and the mean HIV viral load, available for 30 patients, was 3.8±1.2 log^10^ copies/mm^3^. The demographic characteristics and clinical presentation of the VL patients, grouped by HIV infection status are shown in Table 1. Table 2 presents the treatment details and the main outcomes assessed. Of note, *Leishmania*-HIV co-infected patients had a significantly higher frequency of having experienced a previous VL episode (43.5% versus 4.5%), were more frequently malnourished (60.9% versus 25%) and presented a lower frequency of fever and hepatosplenomegaly (67.4% versus 90.9%) compared with non HIV-infected patients.

**Table 1:** Demographic and clinical variables according to HIV infection status.

	HIV negative (%) n = 44	HIV positive (%) n = 46	p
**Age (mean ± SD), years**	37,1**±**14,0	41,0**±**10,9	0.13
**Sex (male∶female)**	11∶33	11∶35	1.00
**Previous VL episode**	2/44 (4.5)	20/46 (43.5)	0.00
**Malnutrition**	11/44 (25.0)	28/46 (60.9)	0.00
**Median length of illness (IR), days**	60 (30–120)	60 (40–91)	0.14
**Median spleen size** # ** (IR), cm**	6 (4–10)	5 (2–7)	0.02
**Median liver size** § **(IR), cm**	5 (4–7)	4 (2–5)	0.01
**Fever**	39/44 (88.6)	28/46 (60.9)	0.00
**Hepatosplenomegaly**	40/44 (90.9)	31/46 (67.4)	0.01
**Cytopenia**	44/44 (100)	46/46 (100)	1.00
**Jaundice**	14/44 (31.8)	7/46 (15.2)	0.12
**Edema**	15/44 (34)	8/46 (17.4)	0.09
**Hypotension**	4/44 (9.1)	8/46 (17.4)	0.35
**Bleeding**	10/44 (22.7)	9/46 (19.6)	0.79
**Bleeding site**			0.81
**Skin/Mucosa**	6/44 (13.6)	4/46 (8.7)	
**Digestive tract**	3/44 (6.8)	4/46 (8.7)	
**Urinary tract**	1/44 (2.3)	1/46 (2.2)	
**Dyspnea**	8/44 (18.2)	8/46 (17.4)	1.00
**Diarrhea**	5/44 (11.4)	10/46 (21.7)	0.26
**Vomiting**	14/44 (31.8)	11/46 (23.9)	0.48
**Median hemoglobin (IR), g/dL**	8.5 (7.2–9.5)	8.2 (7.2–9.0)	0.47
**Median leukocyte count (IR), cells/L**	1850 (1275–2679)	2000 (1575–2800)	0.14
**Median platelets count (IR), cells/L**	90.000 (61500–115.000)	114.500 (82.750–173.000)	0.00
**Median total bilirubin (IR), mg%**	0.9 (0.6–1.55)	0.6 (0.5–1.1)	0.23
**GOT (IR), IU/L**	79 (40.5–155)	44 (27.2–61.7)	0.00
**Serum creatinine (IR), mg%**	0.9 (0.7–1.1)	0.8 (0.7–1.1)	0.25
**RNI (IR)**	1.3 (1.2–1.4)	1.3 (1–1.5)	0.61
**Serum albumin (IR), mg%**	2.6**±**0.6	2.7**±**0.7	0.62

**SD**: standard deviation **IR**: 25–75% interquartile range **VL**: visceral leishmaniasis.

# measured on physical examination at the left midclavicular line.

§ measured on physical examination at the right midclavicular line.

**Hepatosplenomegaly**: palpable spleen or liver 2 cm over the right costal margin, as measured on physical examination **Cytopenia**: the presence of hemoglobin below 12 g% or a leukocyte count of less than 3500 cells/mm^3^ or a platelet count of less than 120000 cells/mm^3^ **GOT:** glutamate oxaloacetate transaminases

**Table 2:** Treatment and outcomes according to HIV infection status.

	HIV negative (%)	HIV positive (%)	p
**VL therapy**			
**Pentavalent antimony**	11/44 (25)	1/46 (2)	0.00
**Deoxycholate amphotericin B**	25/44 (57)	28/46 (61)	
**Liposomal amphotericin B**	8/44 (18)	17/46 (37)	
**VL therapy switching**	20/44 (45.5)	11/46 (23.9)	0.05
**Clinical features during VL treatment**			
**Antibacterial therapy use**	19/44 (43.2)	21/46 (45.7)	0.83
**Febrile neutropenia episode**	14/44 (32.8)	15/46 (32.6)	1.00
**Transfusion of blood components**	23/44 (52.3)	19/46 (41.3)	0.39
**Intensive care unit admission**	9/44 (20.5)	6/46 (13.0)	0.40
**Mechanical ventilator use**	6/44 (13.6)	5/46 (10.9)	0.75
**Hemodialysis requirement**	5/44 (11.4)	4/46 (8.7)	0.74
**Median hospital stay length in days (IR)**	23 (16.3–38.7)	25 (14.5–49.0)	0.40
**Clinical response at end of therapy**			
**Fever response**	37/37 (100)	23/24 (96)	0.82
**Spleen response**	29/34 (85.3)	20/30 (66.7)	0.18
**Hemoglobin response**	15/40 (37.5)	4/43 (9.3)	0.00
**Leukocyte response**	29/36 (80.6)	18/38 (47.4)	0.01
**Platelet response**	27/33 (81.8)	14/33 (42.4)	0.00
**Outcomes during follow-up**			
**Death within 30 days**	2/44 (4.5)	4/46 (8.7)	0.68
**Clinical cure 2 months after treatment**	29/42 (69)	11/46 (24)	0.00
**Clinical cure 6 months after treatment**	39/43 (91)	18/45 (40)	0.00
**Relapse in 6 months**	1/40 (2.5)	14/38 (37)	0.00

**VL**: visceral leishmaniasis **Fever response**: the disappearance of fever at the end of treatment **Hemoglobin response**: patients presenting an increase of 2 g% or more in hemoglobin tax at the end of treatment **Leukocyte response**: patients presenting an increase of 50% or more in their leukocyte count at the end of treatment **Platelet response**: patients presenting an increase of 50% or more in their platelet count at the end of treatment **Spleen response**: a 2 cm or more reduction in spleen size palpation at the end of treatment **Clinical cure:** no death, recurrence, hepatosplenomegaly or hematological abnormalities **IR**: 25–75% interquartile range.

A significant difference between HIV-infected and non HIV-infected patients was observed concerning to all laboratory parameters measured at the end of treatment, as in the relapse rate (Table 2). The rate of regression of fever and reduction of splenomegaly did not differ between the groups. Overall, six deaths were observed within the first month and the conditions associated with early death in the univariate analysis are shown in Table 3. Two patients (2.3%) were lost at a 6 month-follow-up.

**Table 3:** Factors associated with early death (within 1 month after VL diagnosis) from visceral leishmaniasis (univariate analysis).

Variables	Death within 1 month (%)		p value
Univariate analysis	No	Yes	
**Severe neutropenia**	26/84 (30.9)	4/6 (66.7)	0.09
**Severe thrombocytopenia**	10/84 (11.9)	3/6 (50)	0.04
**Bleeding**	14/84 (16.7)	5/6 (83.3)	0.00
**Edema**	19/84 (22.6)	4/6 (66.7)	0.03
**Jaundice**	18/84 (21.4)	4/6 (66.7)	0.14
**Dyspnea**	13/84 (15.5)	4/6 (66.7)	0.01
**HIV infection**	42/84 (50)	4/6 (66.7)	0.68

**Severe neutropenia: a** neutrophil count of less than 500 cells/mm^3^ **Severe thrombocytopenia: a** platelet count of less than 50000 cells/mm^3^.

In the univariate analysis (Table 4), ten variables associated with a poor outcome in VL at 6 months (with a p value<0.2) were selected for inclusion in the multivariate logistic regression model (Table 5). HIV infection, bleeding and a previous VL episode were independent predictors of this unfavorable outcome in the final model. Details about treatment switching can be observed in Table 6.

**Table 4:** Factors associated with a poor outcome in VL (at 6 months after VL diagnosis) in the univariate analysis.

Variables	VL Clinical outcome of VL (%)		p value
	Cure	Poor outcome	
**HIV infection**	18/57 (32)	27/31 (87)	0.00
**Drug abuse** §	8/57 (14)	13/31 (42)	0.00
**Alcohol abuse**	23/57 (40)	22/31 (71)	0.01
**Comorbidity**	12/57 (21)	13/31 (42)	0.09
**Malnutrition**	20/57 (35)	17/31 (58)	0.12
**Bleeding**	9/57 (16)	10/31 (32)	0.10
**Hypotension**	5/57 (9)	7/31 (23)	0.10
**Previous VL episode**	15/31 (71)	6/21 (28)	0.00
**Median percentage of neutrophils (IR)**	48 (42–56)	59 (49–68)	0.00
**Median platelet count (IR), cells/L**	9.9(6.8–12.6×10^4^)	10.1(5.5–16.6×10^4^)	0.19

**VL**: visceral leishmaniasis **IR**: 25–75% interquartile range.

§ in all cases, refers to the use of inhaled drugs derived from cocaine.

**Table 5:** Variables that remained in the final logistic regression model and were associated with a poor outcome in VL (at 6 months after VL diagnosis).

Variables	Odds ratio	95% CI	p value
**Previous VL episode**	8.1	1.6–39.7	0.01
**Bleeding**	8.1	1.5–44.7	0.01
**HIV infection**	10.1	2–51	0.00

Hosmer-Lemeshow goodness-of-fit test. p = 0.24.

**Table 6:** Treatment switching and adverse events observed during initial drug treatment of VL.

Initial VL therapy (patients who initially received drug/patients who switched medication)	Second VL drug therapy (patients)	Reason for change in therapy drug (patients)	Adverse events during initial VL therapy (patients)
**Pentavalent antimony (12/7)**	deoxycholate amphotericin B (6)	toxicity (5); disease severity (1)	renal (1), hepatic (1), and cardiac (1) dysfunction; adverse reactions during infusion (1)
	liposomal amphotericin B (1)	impairment of renal function (1)	renal dysfunction (1)
**Deoxycholate amphotericin B (53/24)**	pentavalent antimony (1)	outpatient treatment using intramuscular medication (1)	none
	liposomal amphotericin B (23)	toxicity (23)	renal (15), hepatic (3), and muscular (1) dysfunction; adverse reactions during infusion (8); electrolytic abnormalities (10); phlebitis (3)
**Liposomal amphotericin B (25/0)**	no patient needed to switch VL therapy		

## Discussion

Despite the high number of reported cases of HIV-related VL, certain aspects of its epidemiology, clinical features and management remain unknown. In addition, few comparative clinical studies on the disease in HIV-infected and non-HIV-infected patients have been reported [14]–[16]. The tertiary hospital where this study was conducted is a referral center in Minas Gerais state for the treatment of HIV infected patients, which certainly explains the high percentage of coinfected patients herein presented, differently from the national rate of around 6% [17]. In the present study, the clinical presentations of VL were similar in HIV-infected and non-infected patients except, as shown by others, fever and hepatosplenomegaly were significantly more common among immunocompetent patients [18]–[22]. Consistent with this finding, the frequency and magnitude of hepatosplenomegaly were significantly lower in patients coinfected by HIV, which has been associated with a deficit in the proliferative response of mononuclear cells in these organs [23]. Because fever and splenomegaly are the two main markers of VL diagnosis, this shift in the clinical presentation of VL may represent a challenge for initial diagnostic workup in areas where VL is spreading.

Thrombocytopenia was more pronounced in immunocompetent patients (p = 0.005), as observed in Ethiopia [16]. Among the numerous actors involved in immune activation and inflammation during HIV infection, activated platelets are inadequately considered [24]. However, platelets are the major source of circulating soluble CD40 ligand, a master immune activator. It has been shown that platelets are an important effector cell of the immune response, able to produce potent inflammatory cytokines in response to exposure to various antigens, including HIV [25]. Although platelet activation during the inflammatory response has been described in terms of functional, but not quantitative aspects, the difference in platelet counts between VL patients (with and without HIV infection) may reflect the activation of a different immune response related to viral presence. Additional research is needed to study this finding.

The drug choice for VL treatment in HIV-infected and uninfected patients reflected the current therapeutic recommendation in Brazil during the study period [10]. In Brazil, pentavalent antimonial drugs are still the first choice for the treatment of VL in non HIV-infected patients due to these drugs proven therapeutic efficacy. Amphotericin B is reserved for patients at extremes of age, patients with signs of clinical severity and patients with comorbidities. Only recently liposomal amphotericin became routinely indicated for the treatment and prophylaxis of VL in HIV co-infected patients. The impact of that change in the Brazilian treatment policy should be evaluated in the near future.

Considering clinical cure as the absence of hepatosplenomegaly on physical examination, the disappearance of fever and the normalization of all hematologic parameters, the analysis showed that 69% and 91% immunocompetent patients were cured at 2 and 6 months after treatment, respectively, compared with only 24% and 40% of HIV-coinfected patients, respectively. Furthermore, we may have underestimated treatment failure rates because a parasitological test of cure is not routinely performed. The VL relapse rate also differed significantly between the two groups: it was 2.5% among immunocompetent individuals (only 1 case), in contrast to 37% among HIV-infected patients. It is important to note that according to the local routine, amphotericin B twice a month is offered as secondary prophylaxis for all patients with a CD4 lymphocyte count lower than 350 cell/mm^3^. A more detailed analysis of factors related to relapse is currently in progress. At the moment, it is possible to observe that many of these relapse episodes occurred under regular use of amphotericin B prophylaxis. In turn, HAART adherence rate is very low in our setting. These findings confirm that patients with HIV-VL coinfection had poorer response rates to antileishmanial treatment, similar to what has been shown by others [26]–[28].

Overall, six deaths were observed within the first month after VL diagnosis. This small number of fatal events prevents us from performing a more comprehensive analysis of the factors related to mortality. However, in Table 3 the conditions associated with a fatal outcome in the univariate analysis are shown. Regardless of the initial drug used in treatment (Table 2), it can be observed that the fatality rate was 8.7% (4/46) for HIV-infected patients, compared with 4.4% (2/44) for HIV uninfected patients. As noted by others, mortality is particularly high among HIV patients treated with antimony derivatives [3], [28], [29]. The World Health Organization (WHO) recommends liposomal amphotericin B as the treatment of choice in coinfected patients [30], although comparative studies between different drugs or different formulations of amphotericin are scarce. In this observational study, the choice of drug for treatment was directly influenced by the severity of disease, according to Brazilian guidelines, which precludes any analysis of outcomes according to treatment. The conditions related to a poor outcome at 6 months were HIV infection, bleeding and a previous VL episode. All three, in addition to other conditions, have already been reported as related to death or severity of VL in three studies from Africa [31]–[33] and in several other studies from Latin America [5], [6], [34]–[39]. Among HIV-uninfected patients, a higher percentage of switching the drug during VL treatment was observed. This phenomenon was due to the large number of patients in this group who began treatment with derivative antimony or amphotericin B deoxycholate, drugs associated with recognized toxicity.

In our analysis, in contrast to the findings of other studies, age was not a determinant of death. This observation possibly reflects the high concentration of young adults studied herein, given the current epidemiology of VL in our urban and recently endemic region [40]. Additionally, we did not identify the length of the disease, diarrhea or vomiting as factors associated with death. It is important to note that our hospital is located in an urban area with relatively good public health coverage. There is a hierarchical system for the referral of patients, which reduces delays in care. Additionally, in contrast to other series, in our experience, HIV infection was not associated with a high risk of death. This finding may partly be due to the non-use of antimony-based treatment in coinfected patients in our setting.

In conclusion, despite slow response and a low rate of normalization of clinical parameters, our data demonstrate that it is possible to achieve levels of VL mortality among HIV-infected individuals that are close to the levels observed in immunocompetent patients by avoiding antimony derivatives use and by providing the minimum conditions for the monitoring and treatment of complications and toxicities.
