# Correction: *Leishmania*-HIV Co-infection: Clinical Presentation and Outcomes in an Urban Area in Brazil

**DOI:** 10.1371/journal.pntd.0003653

**Published:** 2015-03-25

**Authors:** The PLOS Neglected Tropical Diseases Staff

There is an error in Table 1. The values in line 2, Sex (male: female), are incorrect. Please see the table below for the correct values.

**Table 1:** Demographic and clinical variables according to HIV infection status.

	HIV negative (%)n = 44	HIV positive (%)n = 46	p
**Age (mean ± SD), years**	37,1**±**14,0	41,0**±**10,9	0.13
**Sex (male: female)**	34:10	38:8	0.60
**Previous VL episode**	2/44 (4.5)	20/46 (43.5)	0.00
**Malnutrition**	11/44 (25.0)	28/46 (60.9)	0.00
**Median length of illness (IR), days**	60 (30–120)	60 (40–91)	0.14
**Median spleen size# (IR), cm**	6 (4–10)	5 (2–7)	0.02
**Median liver size§ (IR), cm**	5 (4–7)	4 (2–5)	0.01
**Fever**	39/44 (88.6)	28/46 (60.9)	0.00
**Hepatosplenomegaly**	40/44 (90.9)	31/46 (67.4)	0.01
**Cytopenia**	44/44 (100)	46/46 (100)	1.00
**Jaundice**	14/44 (31.8)	7/46 (15.2)	0.12
**Edema**	15/44 (34)	8/46 (17.4)	0.09
**Hypotension**	4/44 (9.1)	8/46 (17.4)	0.35
**Bleeding**	10/44 (22.7)	9/46 (19.6)	0.79
**Bleeding site**			0.81
**Skin/ Mucosa**	6/44 (13.6)	4/46 (8.7)		
**Digestive tract**	3/44 (6.8)	4/46 (8.7)		
**Urinary tract**	1/44 (2.3)	1/46 (2.2)		
**Dyspnea**	8/44 (18.2)	8/46 (17.4)	1.00
**Diarrhea**	5/44 (11.4)	10/46 (21.7)	0.26
**Vomiting**	14/44 (31.8)	11/46 (23.9)	0.48
**Median hemoglobin (IR), g/dL**	8.5 (7.2–9.5)	8.2 (7.2–9.0)	0.47
**Median leukocyte count (IR), cells/L**	1850 (1275–2679)	2000 (1575–2800)	0.14
**Median platelets count (IR), cells/L**	90.000 (61500–115.000)	114.500 (82.750–173.000)	0.00
**Median total bilirubin (IR), mg%**	0.9 (0.6–1.55)	0.6 (0.5–1.1)	0.23
**GOT (IR), IU/L**	79 (40.5–155)	44 (27.2–61.7)	0.00
**Serum creatinine (IR), mg%**	0.9 (0.7–1.1)	0.8 (0.7–1.1)	0.25
**RNI (IR)**	1.3 (1.2–1.4)	1.3 (1–1.5)	0.61
**Serum albumin (IR), mg%**	2.6**±** 0.6	2.7 **±** 0.7	0.62

**SD**: standard deviation **IR**: 25–75% interquartile range **VL**: visceral leishmaniasis **#** measured on physical examination at the left midclavicular line **§** measured on physical examination at the right midclavicular line **Hepatosplenomegaly**: palpable spleen or liver 2 cm over the right costal margin, as measured on physical examination **Cytopenia**: the presence of hemoglobin below 12 g% or a leukocyte count of less than 3500 cells/mm^3^ or a platelet count of less than 120000 cells/mm^3^ **GOT**: glutamate oxaloacetate transaminases
